# Supplementary material for: Single cell RNA analysis uncovers the cell differentiation and functionalization for air breathing of frog lung
Source: Commun Biol. 2024 May 30;7:665. doi: 10.1038/s42003-024-06369-1 (PMC11139932; doi:10.1038/s42003-024-06369-1)
Supplement: Supplementary file 2 — Supplementary Information [file 42003_2024_6369_MOESM2_ESM.pdf]

Supplementary Table 1 scRNA-seq sequence statistics and quality control

Sample	Number of Reads	Valid Barcodes	Sequencing Saturation	Q30 Bases in Barcode	Q30 Bases in RNA Read	Q30 Bases in UMI
s41_1	728,842,980	96.9%	49.6%	95.4%	92.9%	93.0%
s41_2	907,269,367	97.0%	55.3%	95.4%	92.5%	93.1%
s44_1	677,232,117	96.3%	74.0%	95.7%	93.2%	94.2%
s44_2	384,350,669	96.4%	61.1%	95.6%	92.5%	94.2%
sub-adult_1	316,916,715	95.9%	64.3%	94.5%	90.2%	91.9%
sub-adult_2	378,136,705	95.1%	68.4%	94.6%	90.1%	92.5%
adult_1	686,230,510	96.0%	64.1%	95.5%	92.4%	93.2%
adult_2	664,441,366	95.9%	65.9%	95.6%	93.2%	92.9%

Supplementary Table 2 scRNA-seq data mapped to genome and quantification

Sample	Estimated Number of Cells	Mean Reads per Cell	Median Genes per Cell	Reads Mapped to Genome	Reads Mapped Confidently to Genome	Reads Mapped Confidently to Intergenic Regions	Reads Mapped Confidently to Intronic Regions	Reads Mapped Confidently to Exonic Regions	Reads Mapped Confidently to Transcriptome	Reads Mapped Antisense to Gene	Fraction Reads in Cells	Total Genes Detected
s41_1	22,929	31,786	1,047	89.8%	71.6%	23.1%	16.3%	32.1%	30.2%	0.5%	90.0%	24,045
s41_2	27,015	33,583	998	89.9%	73.0%	23.6%	16.5%	32.9%	30.9%	0.5%	91.9%	24,336
s44_1	7,943	85,261	786	90.2%	68.9%	22.3%	13.2%	33.3%	31.3%	0.4%	82.4%	23,935
s44_2	8,055	47,715	767	88.6%	67.5%	22.3%	12.7%	32.5%	30.4%	0.4%	82.3%	23,490
sub-adult_1	8,823	35,919	719	92.9%	84.1%	23.2%	9.8%	51.2%	49.6%	0.3%	89.6%	18,638
sub-adult_2	9,347	40,455	717	91.8%	82.3%	22.6%	9.8%	50.0%	48.3%	0.3%	88.6%	18,873
adult_1	19,535	35,128	725	90.4%	65.6%	19.2%	11.9%	34.5%	32.7%	0.4%	90.8%	21,995
adult_2	18,747	35,442	738	91.3%	67.8%	19.2%	10.9%	37.7%	36.0%	0.4%	92.0%	21,850

Supplementary Table 3 Aggregation and normalization of scRNA-seq data from multiple samples

Sample	Fraction of Reads Kept	Pre-Normalization Total Reads per Cell	Pre-Normalization Confidently Mapped Barcoded Reads per Cell
MF02_1_1	100.0%	31,787	8,503
MF02_1_2	90.6%	33,584	9,385
MF05_1_1	39.6%	85,262	21,468
MF05_1_2	72.5%	47,716	11,732
MF07_2_1	54.1%	35,919	15,711
MF07_2_2	49.9%	40,455	17,037
MF08_2_1	82.7%	35,128	10,285
MF08_2_2	73.5%	35,443	11,574

Supplementary Table 4 Aggregation and normalization of scRNA-seq data from multiple samples

**Quality control indicators**	**Parameter**
Pre-Normalization Total Number of Reads	4,743,420,429
Post-Normalization Total Number of Reads	3,513,409,200
Pre-Normalization Mean Reads per Cell	38,755
Post-Normalization Mean Reads per Cell	28,706
Pre-Normalization Total Number of Reads	4,743,420,429
Post-Normalization Total Number of Reads	3,513,409,200
Pre-Normalization Mean Reads per Cell	38,755
Post-Normalization Mean Reads per Cell	28,706
Estimated Number of Cells	122,394
Fraction Reads in Cells	89.6%
Median Genes per Cell	741
Median UMI Counts per Cell	2,650

Supplementary Table 5 FISH probe sequence

**Gene**	**Probe sequence 5'-3'**
SFTPB	GGTCTCACAGGTCACGTCCTTGTAATGAGGGTCTTC
AHNAK	GCCTCTAAAGTACCACTTGGTGAAGAGTGCGTTG

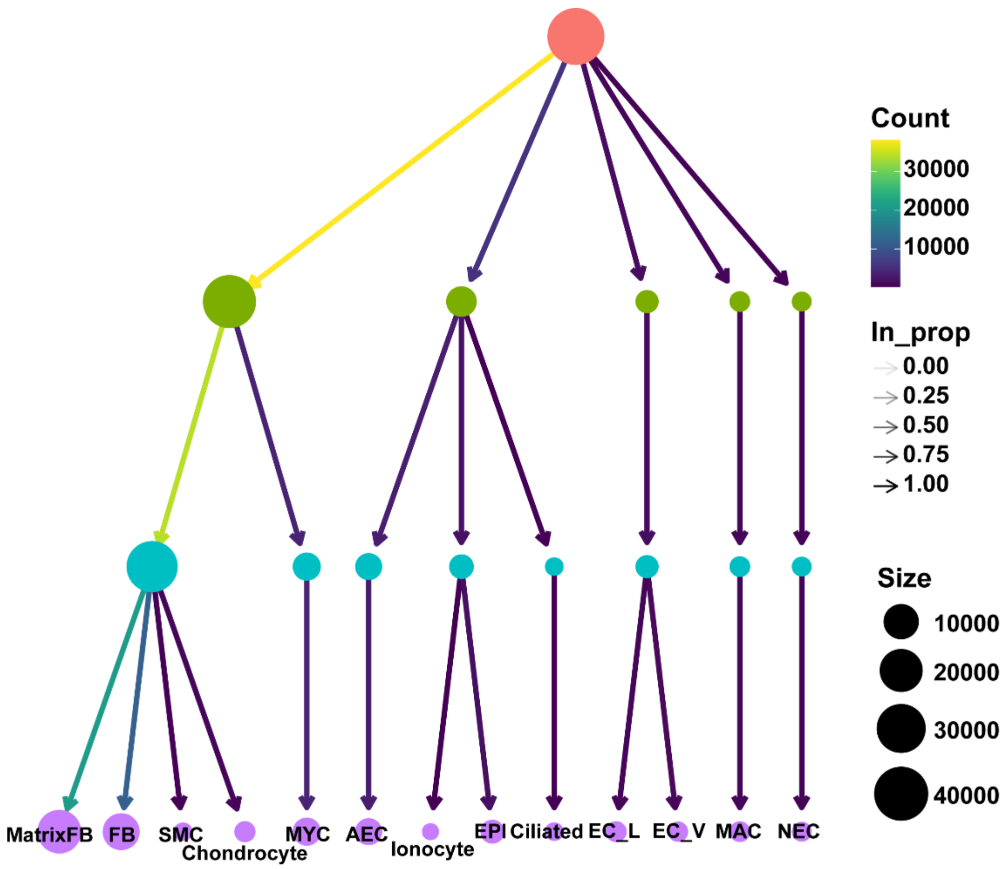

Supplementary Figure 1 Clustertree showing the unbiased clustering results of all of the filtered cells in lung.

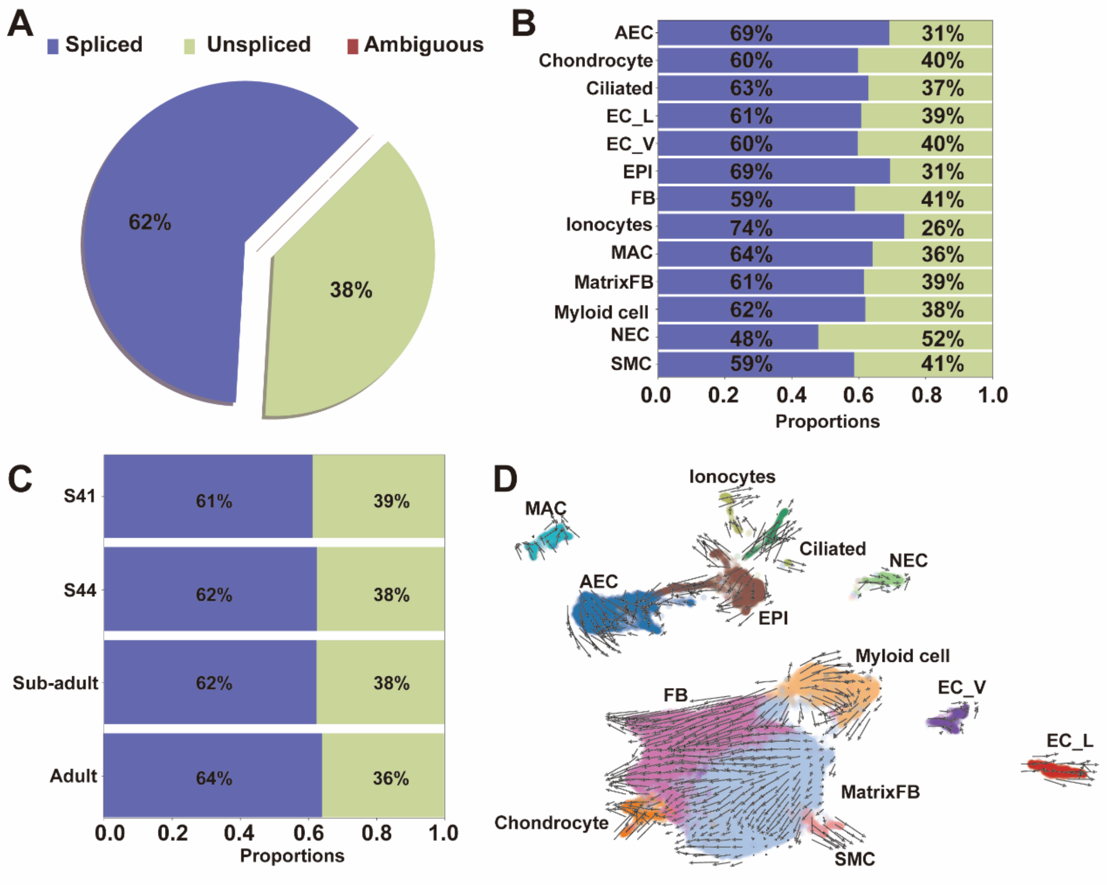

Supplementary Figure 2 Construction of the cellular differentiation trajectory in the developing lung based on the RNA velocity. (A) Pie plot showing the RNA velocity of the developing lung cells. (B, C) Bar plots presenting the RNA velocity of each cell type and developmental stages, respectively. (D) UMAP visualizing the predicted pulmonary cell trajectory based on the RNA velocity.

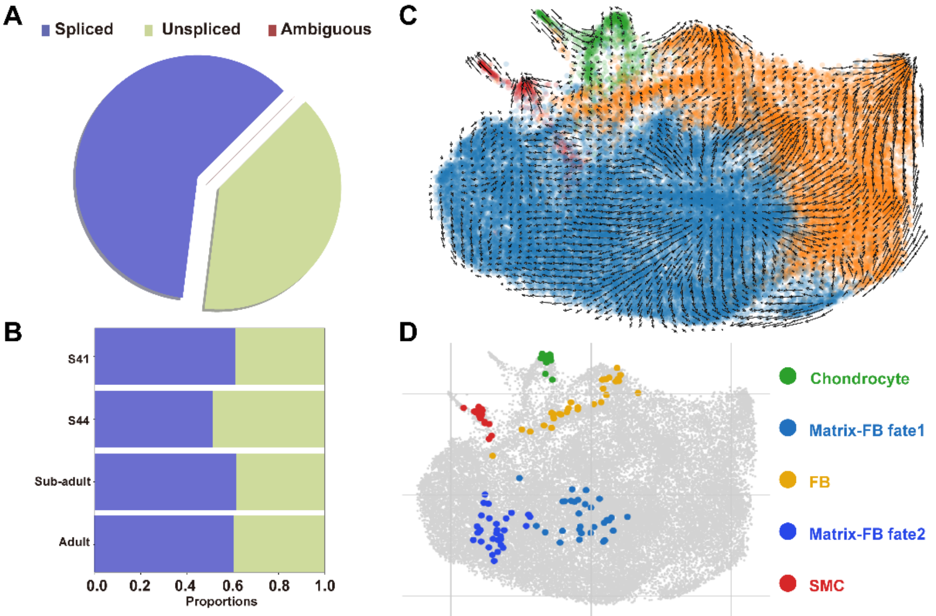

Supplementary Figure 3 Construction of differentiation trajectories of MCs based on the RNA velocity. (A) Pie plot showing the RNA velocity of MCs. (B) Bar plot presenting the cell RNA velocity of each developmental stages, respectively. (C) UMAP visualizing the predicted trajectory of MCs based on the RNA velocity. (D) UMAP visualizing the predicted cell fates of MCs.

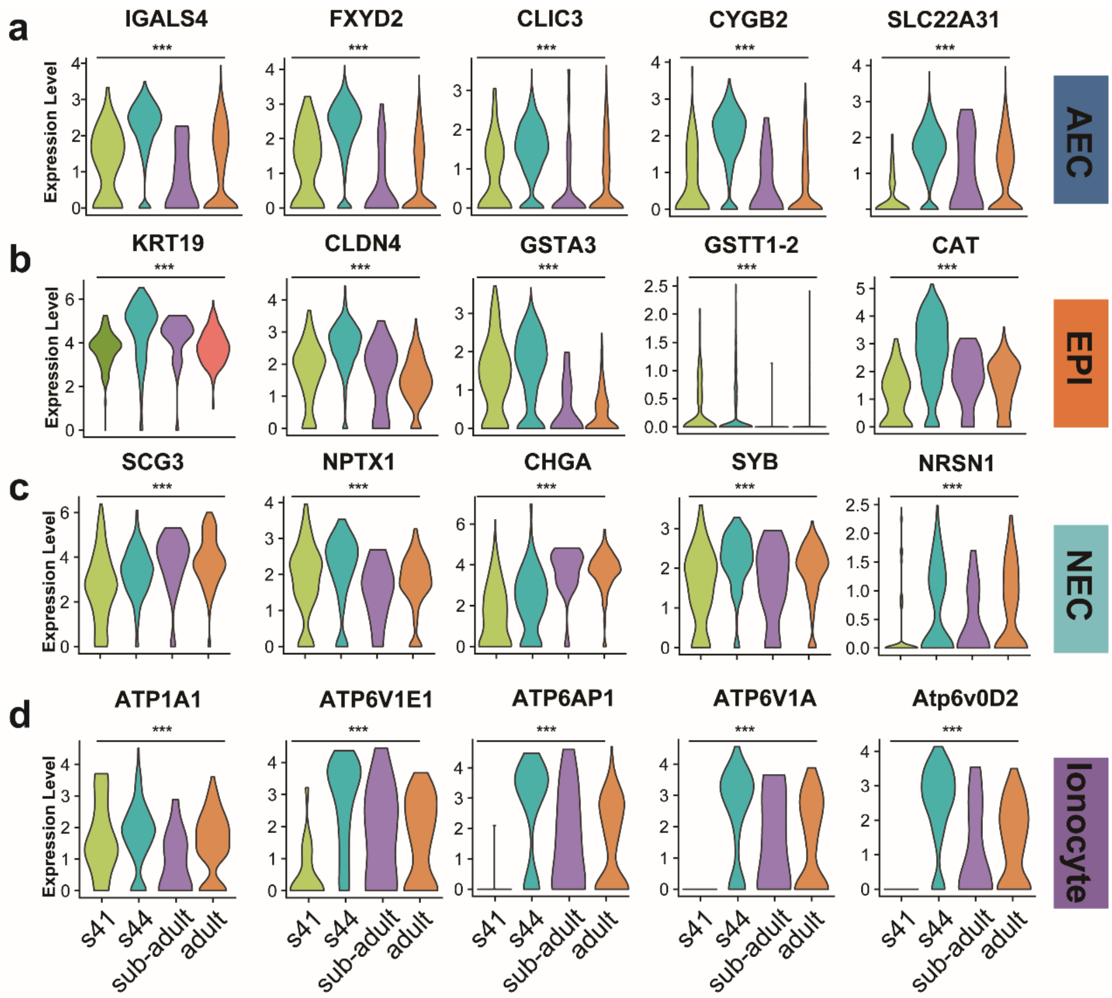

Supplementary Figure 4 The temporal molecular dynamics of pulmonary epithelial cells in 4 developmental stages. (A-D) Violin plots showing the temporal dynamic expression of feature genes in AECs, EPIs, NECs, and Ionocytes, respectively. (***) indicates a significant difference among the expression level in different stages (p value < 0.001).
