# Supplementary material for: Single cell RNA analysis uncovers the cell differentiation and functionalization for air breathing of frog lung
Source: Commun Biol. 2024 May 30;7:665. doi: 10.1038/s42003-024-06369-1 (PMC11139932; doi:10.1038/s42003-024-06369-1)
Supplement: Supplementary file 5 — Reporting Summary [file 42003_2024_6369_MOESM5_ESM.pdf]

nature portfolio

Corresponding author(s): Wei Zhu and Jianping Jiang

Last updated by author(s): May 9, 2024

Reporting Summary

Nature Portfolio wishes to improve the reproducibility of the work that we publish. This form provides structure for consistency and transparency in reporting. For further information on Nature Portfolio policies, see our Editorial Policies and the Editorial Policy Checklist.

Statistics

For all statistical analyses, confirm that the following items are present in the figure legend, table legend, main text, or Methods section.

n/a	Confirmed	
☐	☒	The exact sample size (*n*) for each experimental group/condition, given as a discrete number and unit of measurement
☐	☒	A statement on whether measurements were taken from distinct samples or whether the same sample was measured repeatedly
☐	☒	The statistical test(s) used AND whether they are one- or two-sided<br>*Only common tests should be described solely by name; describe more complex techniques in the Methods section.*
☒	☐	A description of all covariates tested
☐	☒	A description of any assumptions or corrections, such as tests of normality and adjustment for multiple comparisons
☐	☒	A full description of the statistical parameters including central tendency (e.g. means) or other basic estimates (e.g. regression coefficient) AND variation (e.g. standard deviation) or associated estimates of uncertainty (e.g. confidence intervals)
☐	☒	For null hypothesis testing, the test statistic (e.g. *F*, *t*, *r*) with confidence intervals, effect sizes, degrees of freedom and *P* value noted<br>*Give P values as exact values whenever suitable.*
☒	☐	For Bayesian analysis, information on the choice of priors and Markov chain Monte Carlo settings
☐	☒	For hierarchical and complex designs, identification of the appropriate level for tests and full reporting of outcomes
☒	☐	Estimates of effect sizes (e.g. Cohen's *d*, Pearson's *r*), indicating how they were calculated

Our web collection on statistics for biologists contains articles on many of the points above.

Software and code

Policy information about availability of computer code

Data collection	scRNA-seq data of M. fissipes lung are available in the GSA (CRA010691). The source data behind the graphs in the paper are provided in Supplementary Data 1. All the protein sequencing data and annotation of M. fissipes are provided in "Figshare [https://doi.org/10.6084/m9.figshare.25814920.v1]" and "Figshare [https://doi.org/10.6084/m9.figshare.25814923.v1]", respectively. The published scRNA-seq data of human and mouse lung used in this study are available in the GEO database under accession code GSE133747. The published scRNA-seq data of X. laevis were available at "Figshare [https://doi.org/10.6084/m9.figshare.19152839]" .
Data analysis	In this study, we used software general workflow codes, without generating any new code. The R Project for Statistical Computing: R (v4.04); Pre-processing and quality control of scRNA-seq: Cell Ranger (v3.1.0); Integration of scRNAseq datasets: Seurat (v 4.0.2); Pseudotime trajectory analysis: Cellrank 2; Gene ontology (GO) enrichment analysis: KOBAS 3.0.

For manuscripts utilizing custom algorithms or software that are central to the research but not yet described in published literature, software must be made available to editors and reviewers. We strongly encourage code deposition in a community repository (e.g. GitHub). See the Nature Portfolio guidelines for submitting code & software for further information.

Data

Policy information about availability of data

All manuscripts must include a data availability statement. This statement should provide the following information, where applicable:

- Accession codes, unique identifiers, or web links for publicly available datasets
- A description of any restrictions on data availability
- For clinical datasets or third party data, please ensure that the statement adheres to our policy

scRNA-seq data of M. fissipes lung are available in the GSA (CRA010691). The source data behind the graphs in the paper are provided in Supplementary Data 1. All the protein sequencing data and annotation of M. fissipes are provided in "Figshare [https://doi.org/10.6084/m9.figshare.25814920.v1]" and "Figshare [https://doi.org/10.6084/m9.figshare.25814923.v1]", respectively. The published scRNA-seq data of human and mouse lung used in this study are available in the GEO database under accession code GSE133747. The published scRNA-seq data of X. laevis were available at "Figshare [https://doi.org/10.6084/m9.figshare.19152839]" . All other data are available from the corresponding author (or other sources, as applicable) on reasonable request.

Research involving human participants, their data, or biological material

Policy information about studies with human participants or human data. See also policy information about sex, gender (identity/presentation), and sexual orientation and race, ethnicity and racism.

Reporting on sex and gender	Not Applicable
Reporting on race, ethnicity, or other socially relevant groupings	Not Applicable
Population characteristics	Not Applicable
Recruitment	Not Applicable
Ethics oversight	Not Applicable

Note that full information on the approval of the study protocol must also be provided in the manuscript.

Field-specific reporting

Please select the one below that is the best fit for your research. If you are not sure, read the appropriate sections before making your selection.

☒ Life sciences ☐ Behavioural & social sciences ☐ Ecological, evolutionary & environmental sciences

For a reference copy of the document with all sections, see nature.com/documents/nr-reporting-summary-flat.pdf

Life sciences study design

All studies must disclose on these points even when the disclosure is negative.

Sample size	The investigators were not blinded to allocation during the experiments and the outcome assessment
Data exclusions	Filtering criteria for the low-quality cells are provided in the method above
Replication	scRNA-seq were performed with two experimental replicates, all FISH and TEM were performed with at least three experimental replicates.
Randomization	Tissue samples from specific periods were obtained at random.
Blinding	The investigators were not blinded to allocation during the experiments and the outcome assessment.

Reporting for specific materials, systems and methods

We require information from authors about some types of materials, experimental systems and methods used in many studies. Here, indicate whether each material, system or method listed is relevant to your study. If you are not sure if a list item applies to your research, read the appropriate section before selecting a response.

Materials & experimental systems

n/a	Involved in the study
☒	☐ Antibodies
☒	☐ Eukaryotic cell lines
☒	☐ Palaeontology and archaeology
☐	☒ Animals and other organisms
☒	☐ Clinical data
☒	☐ Dual use research of concern
☒	☐ Plants

Methods

n/a	Involved in the study
☒	☐ ChIP-seq
☒	☐ Flow cytometry
☒	☐ MRI-based neuroimaging

Animals and other research organisms

Policy information about studies involving animals; ARRIVE guidelines recommended for reporting animal research, and Sex and Gender in Research

Laboratory animals	Microhyla fissipes: stage 41, 44, sub-adult, and adult
Wild animals	The adults M. fissipes frogs used in this work were the parents of the tadpoles and subadults above, which were collected from farmlands (E 103.459885°, N 30.744614°, 701 m) located in Shifang City, Sichuan Province, China.
Reporting on sex	Sex was not considered in study design
Field-collected samples	egg clutches (ranging from 200 to 500 eggs) of M. fissipes were obtained in lab and placed into 12 aquatic containers (length 42 × width 30 × depth 10 cm, water depth = 5 cm) and hatched (water temperature 25 ± 0.5 °C, light/dark = 12:12 h, lights on at 7:00 h, off at 19:00 h). The hatched tadpoles were fed with the solution of boiled chicken egg yolk once a day for 2 days. Tadpoles were next fed with spirulina powder (China National Salt Industry Corporation) once a day, and water was replaced every 2 days.
Ethics oversight	All procedures applied for this study were approved by the Institutional Ethics Committee of Animal Ethical and Welfare Committee of Chengdu Institute of Biology, Chinese Academy of Sciences (permit: CIB20190201), and all methods were carried out in accordance with the Code of Practice for the Care and Handling of animal guidelines. The study is reported in compliance with the ARRIVE guidelines.

Note that full information on the approval of the study protocol must also be provided in the manuscript.

Plants

Seed stocks	Not Applicable
Novel plant genotypes	Not Applicable
Authentication	Not Applicable
